# Nitrogen reduction in basal fertilization enhances soil physicochemical properties and reshapes microbial community structure to alleviate tobacco bacterial wilt

**DOI:** 10.3389/fmicb.2025.1704525

**Published:** 2026-01-16

**Authors:** Chaojun Shi, Xiufang Yang, Zhengfeng Gao, Yao Liu, Yuqiu Yan, Lijie Chen, Jinhui Jiang, Honglin Zhang, Neng Yang, Jindan Wang, Yinbiao Zhou, Jiahao Jia, Yuxiang Bai, Fen Lv

**Affiliations:** 1 College of Tobacco Science, Yunnan Agricultural University, Kunming, China; 2 Yongsheng County Branch of Lijiang Tobacco Company, Lijiang, China; 3 College of Agronomy and Biotechnology, Yunnan Agricultural University, Kunming, China

**Keywords:** bacterial wilt, nitrogen reduction, soil microbial community, soil physicochemical properties, sustainable disease control

## Abstract

**Introduction:**

Bacterial wilt caused by *Ralstonia solanacearum* is a devastating soil-borne disease that seriously threatens tobacco yield and quality worldwide. Excessive nitrogen fertilization has been widely implicated in soil microecological imbalance and increased disease incidence; however, the regulatory mechanisms underlying nitrogen reduction remain poorly understood.

**Methods:**

Here, a randomized block field experiment was conducted with four basal nitrogen application levels, including conventional fertilization and 10%, 20%, and 30% nitrogen reduction. Disease incidence, rhizosphere soil physicochemical properties, enzyme activities, and microbial community structure were systematically assessed using biochemical analyses and high-throughput sequencing.

**Results:**

The results showed that moderate nitrogen reduction significantly decreased the rhizosphere abundance of *R. solanacearum*, leading to a marked reduction in disease incidence and severity. With decreasing nitrogen input, soil pH increased, while moderate nitrogen reduction significantly enhanced available nitrogen, phosphorus, and potassium, microbial biomass carbon and phosphorus, and optimized the activities of urease, acid phosphatase, nitrate reductase, and nitrite reductase. Microbial community analysis revealed that nitrogen reduction reshaped community structure, increased *α*-diversity, and enriched beneficial genera such as *Arthrobacter* and *Amycolatopsis*. Redundancy analysis further identified soil pH, microbial biomass carbon, acid phosphatase activity, and soil organic matter as the primary drivers of microbial community shifts.

**Discussion:**

Overall, these findings demonstrate that moderate reduction of basal nitrogen fertilization effectively suppresses tobacco bacterial wilt by improving rhizosphere soil properties and steering microbial community assembly toward a disease-suppressive state. This study provides both a theoretical basis and practical guidance for sustainable tobacco disease management and nitrogen reduction strategies.

## 1 Introduction

Bacterial wilt, caused by *Ralstonia solanacearum*, is widely distributed worldwide, particularly in tropical, subtropical, and some temperate regions. It significantly reduces the yield of Solanaceae crops and results in substantial economic losses (Campos and Ortiz, 2020). In China, the disease is mainly prevalent in the southern and eastern regions, where it poses severe threats to crop cultivation and profitability (Jiang et al., 2017). In tobacco production, bacterial wilt is especially destructive, as it disrupts the vascular tissues of plants, leading to wilting and considerable yield loss (Wang Z. et al., 2022). More alarmingly, with global climate warming, the distribution of bacterial wilt has expanded to higher latitudes, further exacerbating its threat to the secure production of tobacco and other Solanaceae crops. Therefore, the development of efficient control strategies to ensure sustainable and environmentally friendly tobacco production has become an urgent priority.

Among various control approaches, agricultural management represents an effective and eco-friendly strategy, with nutrient management playing a pivotal role. Appropriate nitrogen (N) fertilization can enhance plant resistance to pathogens, thereby improving crop growth and metabolism (Yang et al., 2024). Both the ability of pathogens to utilize nitrogen and the supply level of nitrogen in the soil are critical factors influencing plant defense against infections (Sun et al., 2020). Studies have shown that different nitrogen forms not only provide nutrients to plants but also modulate the progression of bacterial wilt. For instance, nitrate assimilation promotes the adhesion of *R. solanacearum* to roots, colonization in stems, and increases in virulence (Dalsing et al., 2015), whereas ammonium fertilizers can reduce disease incidence by inhibiting pathogen growth and increasing soil pH, thereby suppressing the pathogen (Liu et al., 2016). Liu W. et al. (2024) further reported that excessive nitrogen input significantly elevates bacterial wilt incidence in tomato.

Soil nutrient status strongly influences the composition of rhizosphere microbial communities and is closely linked to soil-borne disease occurrence. Nutrient imbalance can markedly increase disease incidence (Jiao et al., 2024), whereas organic fertilization has been reported to enhance plant disease resistance (Su et al., 2022). Yang et al. (2024) found that excessive nitrate levels reduced the relative abundance of key biocontrol bacteria such as *Bacillus* and *Lysinibacillus*, indirectly facilitating pathogen proliferation. Similarly, Cao et al. (2024) demonstrated that alleviating phosphorus limitation enhanced the suppressive potential of rhizosphere microbiomes, as sufficient phosphorus promoted root exudation and enriched beneficial microbes. In contrast, Zhang H. et al. (2022) revealed that higher total nitrogen and organic carbon levels intensified competition between bacteria and fungi, aggravating yellow leaf disease severity.

Soil microbes play a fundamental role in plant disease suppression and represent the primary determinant of soil-borne disease dynamics (Pedrinho et al., 2024). Suppressive soils are enriched in resistant microorganisms that protect plants through mechanisms such as inducing host resistance, competing with pathogens, or directly inhibiting pathogen activity (Todorović et al., 2023). In diseased rhizospheres, beneficial bacteria such as *Sphingomonas* decline, while pathogen abundance increases, leading to microecological imbalance (Yuan et al., 2019). By contrast, healthy rhizospheres often harbor higher abundances of phages specific to *R. solanacearum*, reducing pathogen populations (Yang et al., 2024). Moreover, microbial diversity is closely linked to disease suppression; soils with higher diversity exhibit broader ecological functions, greater resilience to environmental stress, and improved crop productivity (Liang et al., 2024). In recent years, continuous tobacco monoculture, combined with excessive and unbalanced application of fertilizers and pesticides, has resulted in deteriorating soil health, frequent root and stem diseases, reduced leaf quality, and unsustainable soil utilization. Over-application of chemical nitrogen fertilizers has further caused nitrogen surplus, lower nitrogen use efficiency, and environmental pollution (Chen et al., 2023; Xia et al., 2025). Although numerous studies have investigated the etiology and management of tobacco bacterial wilt, the specific role of nitrogen levels in disease occurrence remains insufficiently explored.

To address this gap, the present study established field plots in areas with long-term high bacterial wilt incidence, applying different basal nitrogen levels. By assessing disease incidence and severity, rhizosphere soil nutrient profiles, and microbial community dynamics in healthy versus diseased plants, we aimed to elucidate the regulatory role of basal nitrogen fertilization on bacterial wilt occurrence. Furthermore, we sought to identify key soil nutrients and microbial interactions associated with disease suppression. This work provides theoretical underpinnings for optimizing nitrogen fertilization strategies and developing sustainable, nutrient-based agricultural approaches for the green control of tobacco bacterial wilt.

## 2 Materials and methods

### 2.1 Experimental site

The field experiment was conducted in Dongtun Township, Xixiu District, Anshun City, Guizhou Province, China (26°10′40″ N, 106°14′6″ E, 1222.7 m a.s.l.), a region with a long history of severe tobacco bacterial wilt incidence. Before this experiment, the field had been under continuous tobacco cultivation for 6 years, and no soil fumigation was applied. The soil type was yellow soil with uniform fertility and flat terrain. The basic soil physicochemical properties were as follows: pH 5.48, total nitrogen (TN) 2.15 g·kg^−1^, soil organic matter (SOM) 35.96 g·kg^−1^, available phosphorus (AP) 126.74 mg·kg^−1^, available potassium (AK) 227.18 mg·kg^−1^, and alkali-hydrolyzable nitrogen (AN) 173.65 mg·kg^−1^.

### 2.2 Experimental design and soil sampling

#### 2.2.1 Experimental design

A randomized block design was employed with four basal nitrogen (N) application levels: 75, 67.5, 60, and 52.5 kg·hm^−2^, corresponding to the conventional rate (CK) and 10, 20, and 30% reductions of basal N application, respectively. For each N level, rhizosphere soils were collected from both diseased and healthy plants, resulting in eight treatments. Each treatment had three replicates, with at least 150 plants per replicate. The tobacco cultivar used was Yunyan 87, planted at a spacing of 110 cm × 55 cm, with border rows as buffers. Fertilizers included a tobacco-specific compound fertilizer (N-P₂O₅-K₂O = 10–11-24), superphosphate (P₂O₅ ≥ 12%), and potassium sulfate (K₂O ≥ 52%). Nitrogen reduction treatments were based on local conventional fertilization rates, with phosphorus and potassium replenished using superphosphate and potassium sulfate. Topdressing was conducted according to local agronomic practices, and all other management measures, including irrigation, were kept consistent across treatments and followed the regional guidelines for high-quality tobacco production, in order to minimize management-related effects on nutrient availability, microbial dynamics, and disease development.

#### 2.2.2 Soil sampling

Soil samples were collected one week after tobacco topping. This time point was selected because root exudation is relatively stable after topping, and the symptoms of bacterial wilt are clearly distinguishable across treatments, ensuring comparability and reproducibility. For each treatment, rhizosphere soils were sampled separately from diseased and healthy plants, with three replicates per treatment. In each replicate, three plants with uniform growth (healthy) or comparable disease severity (diseased) were selected. After removing debris from roots, soils within 2 mm of the root surface were detached by shaking and pooled as one replicate. Stones and residues were removed, and the soil was divided into two portions: one was transferred into 10 mL cryogenic tubes, flash-frozen in liquid nitrogen, and stored at −80 °C for DNA extraction, while the other was stored at 4 °C for physicochemical and enzyme activity analyses. Soils were classified into diseased soil and non-diseased soil based on field symptom observation and pathogen abundance. Diseased soil was collected from plots showing typical bacterial wilt symptoms on tobacco plants, whereas non-diseased soil was obtained from plots cultivated with tobacco but showing no visible wilt symptoms during the growing season. The classification was further supported by quantitative PCR analysis, which confirmed a substantially higher abundance of *Ralstonia solanacearum* in diseased soils than in non-diseased soils.

### 2.3 Measurements and analytical methods

#### 2.3.1 Soil physicochemical properties

Soil pH was measured in a 2.5:1 soil-to-water suspension using a potentiometric method (Arunrat et al., 2022). SOM was determined by potassium dichromate oxidation (Gelman et al., 2012). TN was measured using the potassium dichromate–sulfuric acid digestion method (Flowers and Bremner, 1991). AN was determined by the micro-diffusion titration method (Rukun, 1999). AK was extracted with NH₄OAc and determined by flame photometry (Li J. et al., 2021). AP was measured using the sodium bicarbonate-molybdenum antimony colorimetric method (Yuan et al., 2020). Soil microbial biomass carbon and nitrogen (MBC, MBN) were determined using the chloroform fumigation-extraction method with 0.5 mol·L^−1^ K₂SO₄ (Witt et al., 2000). Microbial biomass phosphorus (MBP) was determined by chloroform fumigation–extraction with 0.5 mol·L^−1^ NaHCO₃ (pH 8.5) (Brookes et al., 1982; Hedley and Stewart, 1982).

#### 2.3.2 Soil enzyme activities

The activities of urease (URE), nitrate reductase (SNR), nitrite reductase (SNIR), and acid phosphatase (ACP) were determined using commercial assay kits (Suzhou Grace Biotechnology Co., Ltd., Suzhou, China).

#### 2.3.3 Soil microbial community analysis

Soil DNA was extracted using the HiPure Soil DNA Kit (D3142, Magen Biotechnology Co., Ltd., Guangzhou, China). DNA quality and concentration were assessed by 1% agarose gel electrophoresis. DNA purity was further determined using a NanoDrop 2000 spectrophotometer, and samples with A260/280 values of 1.8–2.0 and A260/230 ≥ 1.8 were used for downstream sequencing analyses. Diluted genomic DNA was used as a template for PCR amplification with barcode-tagged specific primers targeting selected sequencing regions, using high-fidelity polymerase (New England Biolabs, USA). PCR products were purified with AMPure XP beads and quantified using Qubit 3.0 (Thermo Fisher Scientific). A second round of amplification was followed by purification with AMPure XP beads, and quantification was performed on an ABI StepOnePlus Real-Time PCR System (Life Technologies, USA). Sequencing was conducted on an Illumina NovaSeq 6,000 platform (PE250 mode). Low-quality reads were filtered using USEARCH, paired-end reads were merged into tags, and chimeras were removed during clustering to obtain operational taxonomic units (OTUs) and representative sequences.

#### 2.3.4 Quantification of *Ralstonia solanacearum*

DNA was extracted from soil samples using the PowerSoil® DNA Isolation Kit (MoBio Laboratories, USA). The abundance of *R. solanacearum* was quantified by qPCR based on Ct values, and copy numbers were calculated using a standard curve (Supplementary Figure S1). The standard curve exhibited a correlation coefficient (R^2^) greater than 0.99, with amplification efficiencies ranging from 90 to 110%, ensuring reliable quantification.

### 2.4 Data analysis

Data were organized using Excel 2024, and statistical analyses (ANOVA and correlation) were performed with SPSS 26. Figures were generated with Origin 2024. Microbial community analyses were conducted using the OmicShare online platform (https://www.omicshare.com/tools). Microbial co-occurrence networks were analyzed in R 4.2.3 using the “igraph” and “bipartite” packages, and visualized with Gephi 0.10.

## 3 Results

### 3.1 Abundance of *Ralstonia solanacearum* in the rhizosphere

With decreasing basal nitrogen application levels, the overall incidence of tobacco bacterial wilt showed a downward trend, with significant differences among treatments. The 20% nitrogen reduction treatment exhibited the lowest disease incidence, which was 4.88% lower than the control, while the disease index decreased by 2.37 (Supplementary Table S1). The copy number of *R. solanacearum* was significantly higher in diseased soils compared with non-diseased soils. Moreover, nitrogen reduction altered the abundance of *R. solanacearum* under different fertilization regimes. In both diseased and non-diseased soils, the pathogen abundance showed a declining trend, and all nitrogen reduction treatments had lower bacterial copy numbers than the conventional control (Supplementary Table S2).

### 3.2 Rhizosphere soil physicochemical properties and enzyme activities

**Figure 1:**
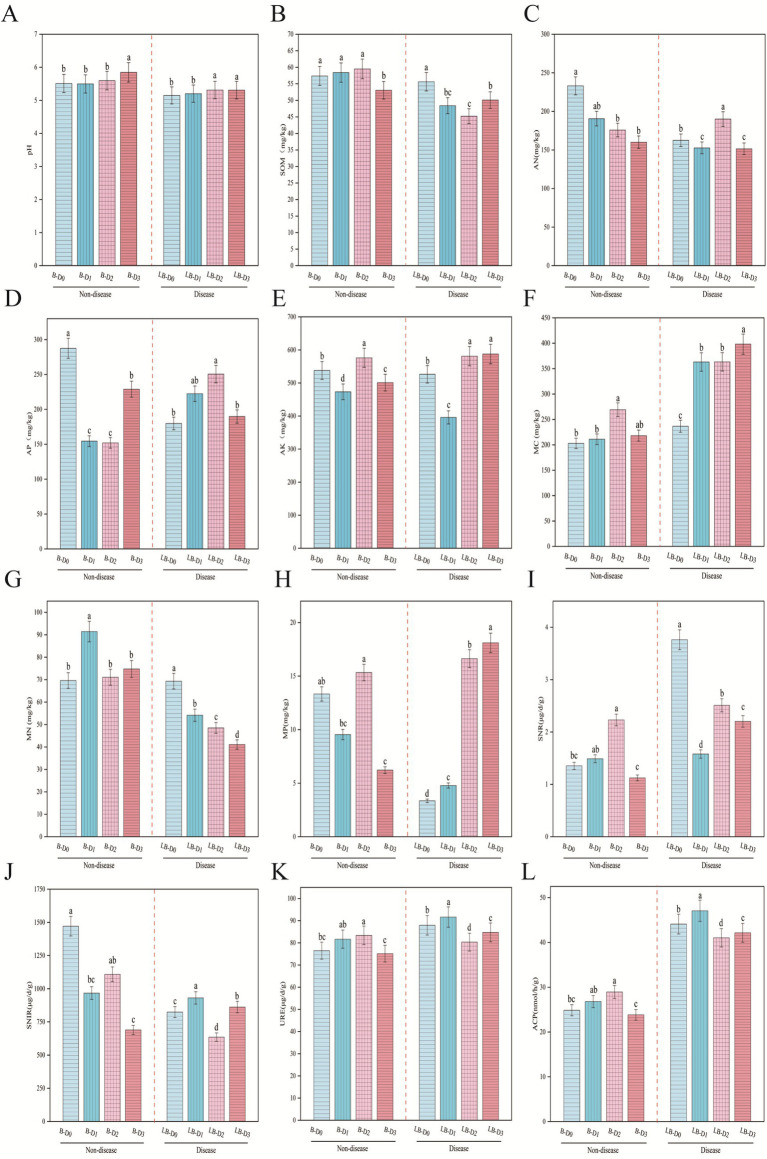
Effects of basal fertilizer nitrogen reduction on soil physicochemical properties and enzyme activities. Panels **(A–H)** represent soil physicochemical properties, and panels **(I–L)** represent soil enzyme activities, including pH, soil organic matter, hydrolyzable nitrogen, available phosphorus, available potassium, microbial biomass C/N/P, nitrate reductase, nitrite reductase, urease, and acid phosphatase, in sequence. Different lowercase letters above the bars indicate significant differences among treatments according to Duncan’s multiple range test (*p* < 0.05; the same applies hereinafter).

Basal nitrogen reduction significantly affected the physicochemical properties and enzyme activities of diseased soils. At the same nitrogen level, diseased soils showed lower pH, soil organic matter (SOM), and microbial biomass nitrogen (MN) compared with non-diseased soils (Figures 1A,B,G), but exhibited higher microbial biomass carbon (MC), soil nitrate reductase (SNR), and acid phosphatase (ACP) contents (Figures 1F,I,L).

In diseased soils, compared with conventional nitrogen application:10% nitrogen reduction significantly decreased MN (Figure 1G) but markedly increased microbial biomass phosphorus (MP) and soil nitrite reductase (SNIR) (Figures 1H,J)0.20% nitrogen reduction significantly reduced SNR, urease (URE), and ACP activities (Figures 1I,K,L), while significantly enhancing available nitrogen (AN) and available phosphorus (AP) (Figures 1C,D)0.30% nitrogen reduction significantly increased available potassium (AK), MP, and SNIR (Figures 1E,H,J). These responses were generally opposite to the corresponding changes observed in non-diseased soils under the same nitrogen treatments.

### 3.3 Changes in soil microbial community *α*-diversity

The rhizosphere soils of diseased tobacco plants exhibited higher bacterial and fungal richness (Chao1) as well as diversity indices (Shannon and Simpson) compared with non-diseased soils (Table 1). With decreasing basal nitrogen application, bacterial richness in both non-diseased and diseased soils followed an initial increase and subsequent decline, although the differences were not statistically significant. In diseased soils, nitrogen reduction significantly decreased the Shannon index while increasing the Simpson index.

**Table 1:** Alpha diversity indices of microbial communities at the OTU level in rhizosphere soils.

Microbial type	Treatment	Chao1	Shannon	Simpson
Bacteria	B-D0	2565.21 ± 83.35a	8.61 ± 0.13a	0.990 ± 0.001ab
	B-D1	2595.94 ± 31.54a	8.59 ± 0.10a	0.988 ± 0.02ab
	B-D2	2666.21 ± 38.88a	8.57 ± 0.18a	0.987 ± 0.003b
	B-D3	2541.76 ± 160.97a	8.64 ± 0.31a	0.992 ± 0.003a
	LB-D0	2553.90 ± 105.68a	8.63 ± 0.03b	0.992 ± 0.001b
	LB-D1	2573.56 ± 102.11a	8.80 ± 0.10a	0.994 ± 0.001a
	LB-D2	2694.20 ± 54.71a	8.88 ± 0.04a	0.994 ± 0.001a
	LB-D3	2661.41 ± 45.33a	8.92 ± 0.07a	0.994 ± 0.001a
Fungi	B-D0	705.09 ± 40.99b	4.83 ± 0.06b	0.911 ± 0.006b
	B-D1	753.31 ± 56.54ab	5.28 ± 0.24a	0.942 ± 0.013a
	B-D2	718.23 ± 29.72ab	5.18 ± 0.23ab	0.927 ± 0.007ab
	B-D3	807.42 ± 65.64a	5.08 ± 0.26ab	0.914 ± 0.020b
	LB-D0	752.09 ± 10.84b	5.40 ± 0.17a	0.944 ± 0.004a
	LB-D1	825.29 ± 15.35a	5.42 ± 0.28a	0.938 ± 0.015a
	LB-D2	812.38 ± 59.44a	5.39 ± 0.28a	0.937 ± 0.012a
	LB-D3	818.83 ± 39.00a	5.61 ± 0.96a	0.946 ± 0.007a

Values followed by different lowercase letters within the same column indicate significant differences among treatments according to Duncan’s multiple range test (*P* < 0.05). Treatments included two soil types, non-diseased soil (B) and diseased soil (LB), combined with four basal nitrogen (N) application levels. For non-diseased soil, treatments were designated as B-D0 (conventional fertilization, CK, 75 kg N·hm^−2^), B-D1 (10% basal N reduction, 67.5 kg N·hm^−2^), B-D2 (20% basal N reduction, 60 kg N·hm^−2^), and B-D3 (30% basal N reduction, 52.5 kg N·hm^−2^). For diseased soil, corresponding treatments were LB-D0 (CK, 75 kg N·hm^−2^), LB-D1 (10% reduction, 67.5 kg N·hm^−2^), LB-D2 (20% reduction, 60 kg N·hm^−2^), and LB-D3 (30% reduction, 52.5 kg N·hm^−2^).

For fungal communities, nitrogen reduction significantly enhanced the Chao1 index in diseased soils. In non-diseased soils, the 10% nitrogen reduction treatment markedly increased both the Shannon and Simpson indices, whereas no significant differences were observed in diseased soils. Reducing nitrogen by 20% can increase bacterial community richness, while a 30% nitrogen reduction significantly enhances fungal community richness.

### 3.4 Changes in soil microbial community *β*-diversity

**Figure 2:**
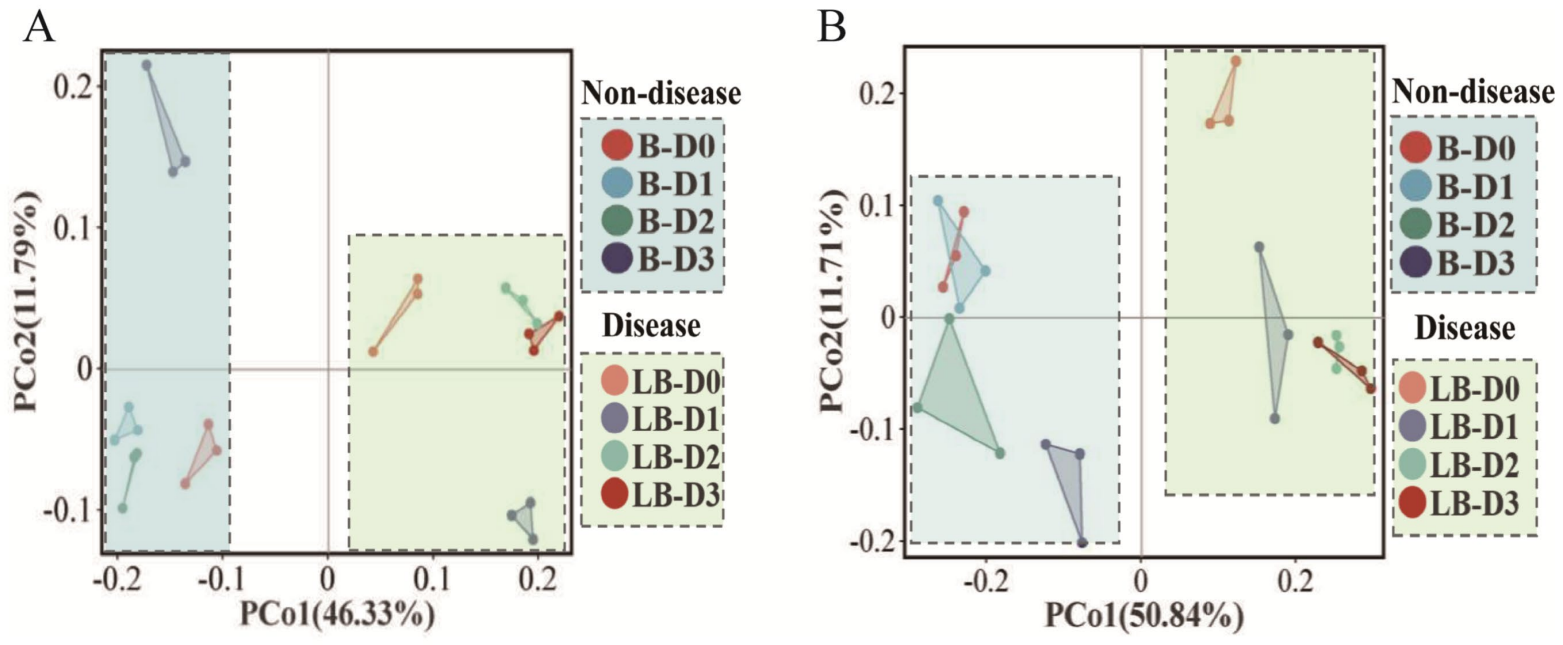
Beta diversity of soil microbial communities. Principal coordinate analysis (PCoA) based on the unweighted UniFrac distance algorithm revealed significant differences among treatments (*p* < 0.05), with Panel **(A)** showing bacterial community structure and Panel **(B)** showing fungal community structure.

Different basal nitrogen levels exerted distinct effects on the microbial community structures of non-diseased and diseased soils (Figure 2). For bacterial communities, PCo1 and PCo2 explained 58.12% of the variation. Samples from non-diseased soils clustered within the second and third quadrants, whereas those from diseased soils were located in the first and fourth quadrants, indicating a clear separation between the two groups (Figure 2A). As nitrogen input decreased, bacterial communities in non-diseased soils became more dispersed across treatments, while those in diseased soils were more closely clustered, suggesting lower species differentiation.

For fungal communities, PCo1 and PCo2 together explained 62.55% of the variation. Non-diseased soil samples were distributed in the second and third quadrants, whereas diseased soil samples were concentrated in the first and fourth quadrants, again revealing distinct community separation (Figure 2B). In non-diseased soils, the conventional treatment and the 10% nitrogen reduction treatment were not clearly separated, while the other treatments diverged along the PCo2 axis. In diseased soils, the 20 and 30% nitrogen reduction treatments clustered closely together, suggesting reduced species differentiation, but were distinctly separated from the conventional treatment along the PCo1 axis.

### 3.5 Composition of soil microbial communities

**Figure 3:**
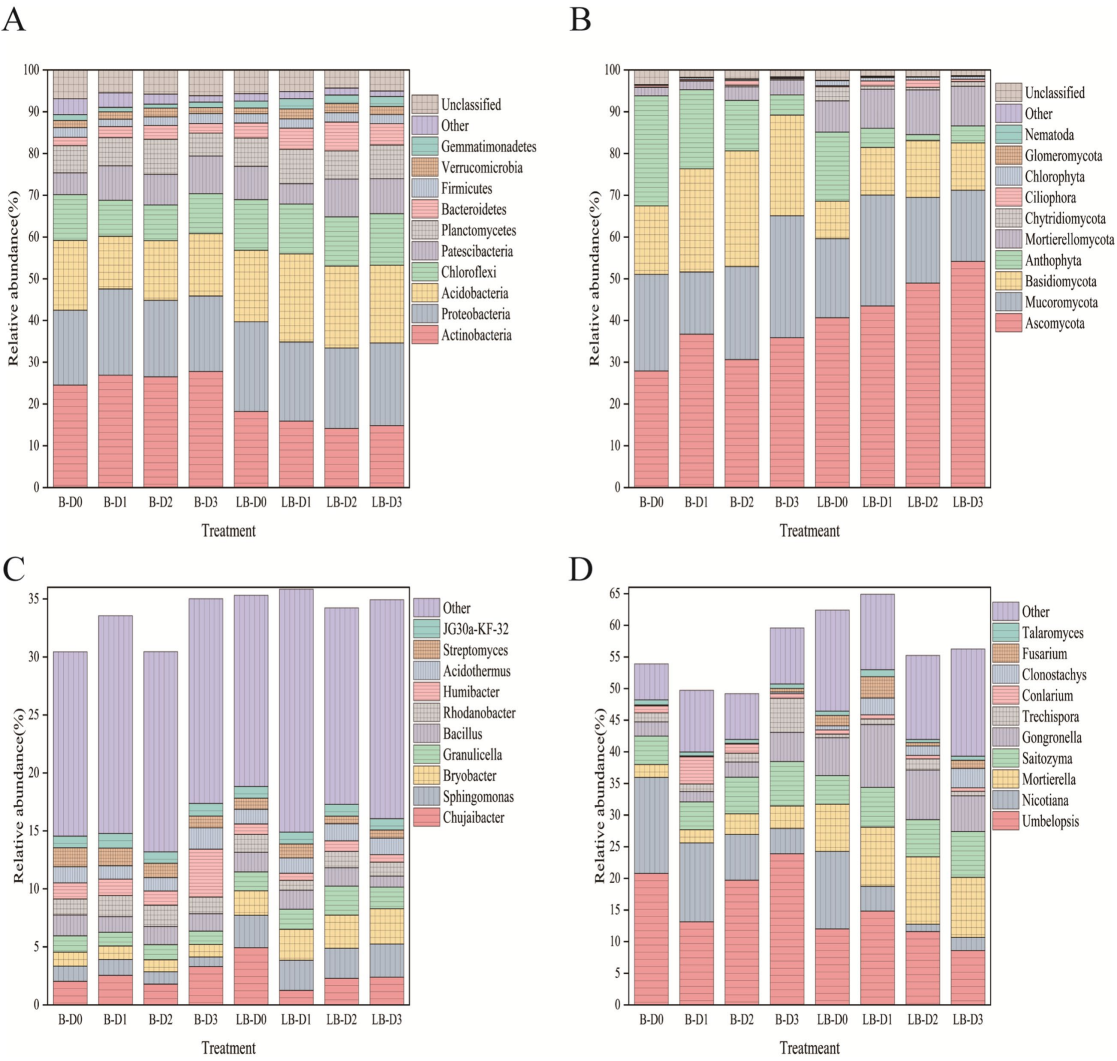
Composition of soil microbial communities. **(A)** Bacterial community composition at the phylum level; **(B)** Fungal community composition at the phylum level; **(C)** Bacterial community composition at the genus level; **(D)** Fungal community composition at the genus level.

Basal nitrogen reduction markedly altered the relative abundances of soil microbial taxa. Compared with non-diseased soils, diseased soils showed decreased abundance of Actinobacteria, whereas Proteobacteria, Acidobacteria, Chloroflexi, Bacteroidetes, and Firmicutes increased (Figure 3A). At the genus level, the relative abundances of *Sphingomonas*, *Bryobacter*, and *Granulicella* were elevated, while *Humibacter* and *Streptomyces* declined (Figure 3C). For fungi, the abundances of Ascomycota and Chytridiomycota increased, and Mucoromycota increased under most treatments except the 10% nitrogen reduction, whereas Basidiomycota, Anthophyta, and Mortierellomycota decreased (Figure 3B). Genera such as *Umbelopsis* declined under all treatments except the 10% nitrogen reduction, while *Nicotiana, Trechispora,* and *Conlarium* decreased; in contrast, *Mortierella, Saitozyma, Gongronella, Clonostachys,* and *Fusarium* increased (Figure 3D).

**Figure 4:**
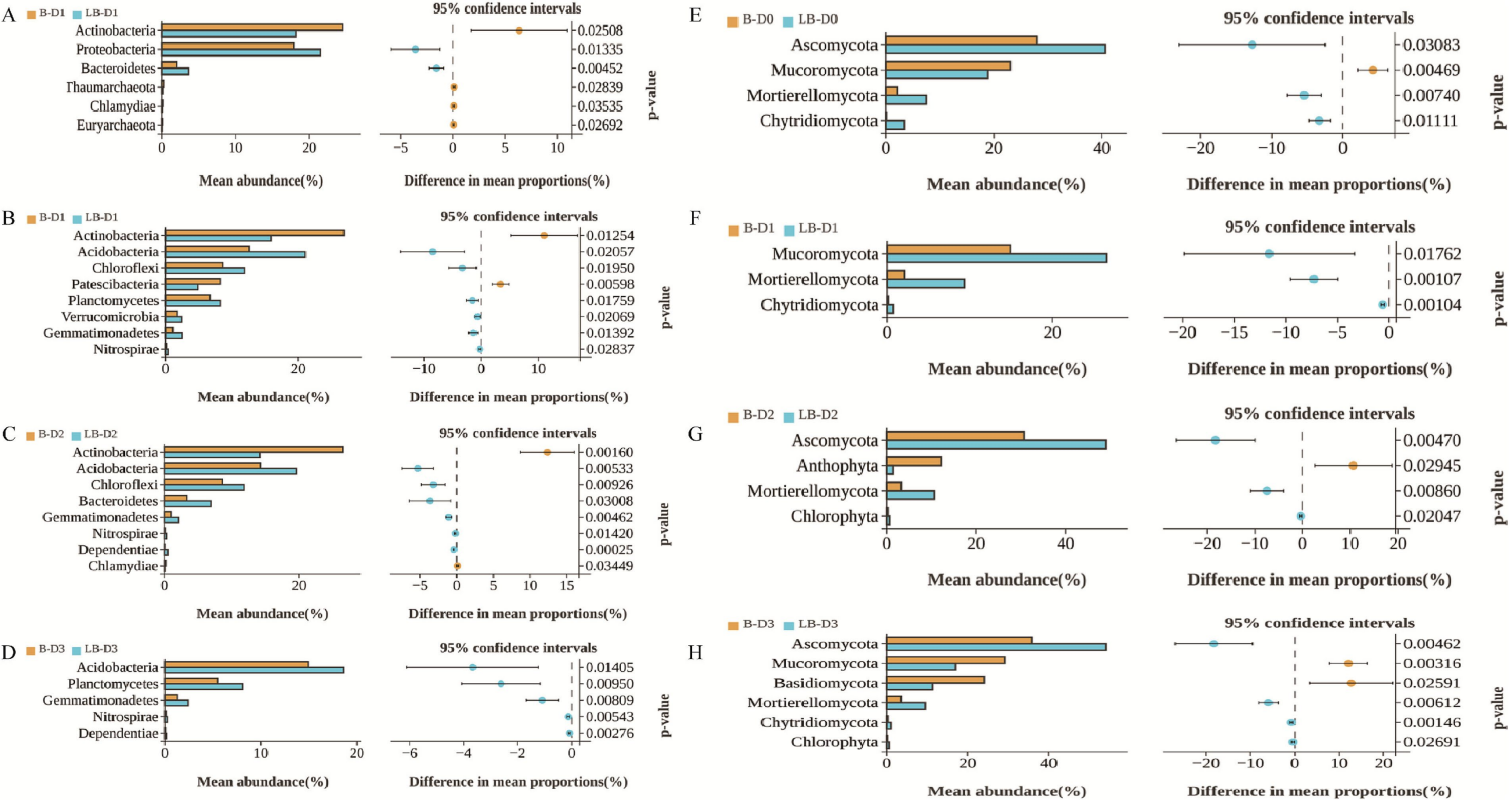
Differential analysis of microbial communities in non-diseased and diseased soils. Taxa with a relative abundance ≥0.1% at the phylum level were selected. Welch’s t-test was applied to compare non-diseased and diseased soils under identical nitrogen levels (*p* = 0.05). Panels **(A–D)** represent bacterial communities under conventional fertilization and 10%, 20%, and 30% nitrogen reduction, while Panels (E–H) represent fungal communities under the same treatments.

Under conventional nitrogen fertilization, Actinobacteria in diseased soils were significantly lower than in non-diseased soils, whereas Proteobacteria and Bacteroidetes were significantly higher (Figure 4A). In fungal communities, Mucoromycota were significantly lower in diseased soils, while Ascomycota, Mortierellomycota, and Chytridiomycota were significantly enriched (Figure 4E).

At 10% nitrogen reduction, Actinobacteria and Patescibacteria in diseased soils were significantly lower, whereas Acidobacteria, Chloroflexi, Planctomycetes, Verrucomicrobia, and Gemmatimonadetes were significantly higher than in non-diseased soils (Figure 4B). Fungal communities in diseased soils exhibited significant enrichment of Mucoromycota, Mortierellomycota, and Chytridiomycota (Figure 4F).

At 20% nitrogen reduction, Actinobacteria were significantly reduced in diseased soils, while Acidobacteria, Chloroflexi, Bacteroidetes, and Gemmatimonadetes were significantly enriched compared with non-diseased soils (Figure 4C). In fungal communities, Anthophyta decreased, whereas Ascomycota, Mortierellomycota, and Chlorophyta increased (Figure 4G).

At 30% nitrogen reduction, diseased soils showed significantly higher abundances of Acidobacteria, Planctomycetes, and Gemmatimonadetes than non-diseased soils (Figure 4D). For fungi, Mucoromycota and Basidiomycota were significantly lower, while Ascomycota and Mortierellomycota were enriched (Figure 4H).

### 3.6 Correlation network analysis of soil microbial communities

In microbial co-occurrence networks, diseased soils exhibited higher values of node number, average degree, average weighted degree, graph density, and average clustering coefficient compared with non-diseased soils, while the number of edges, modularity, and average path length were lower (Supplementary Table S3). These results indicate that the rhizosphere microbial networks became more complex following the occurrence of tobacco bacterial wilt.

**Figure 5:**
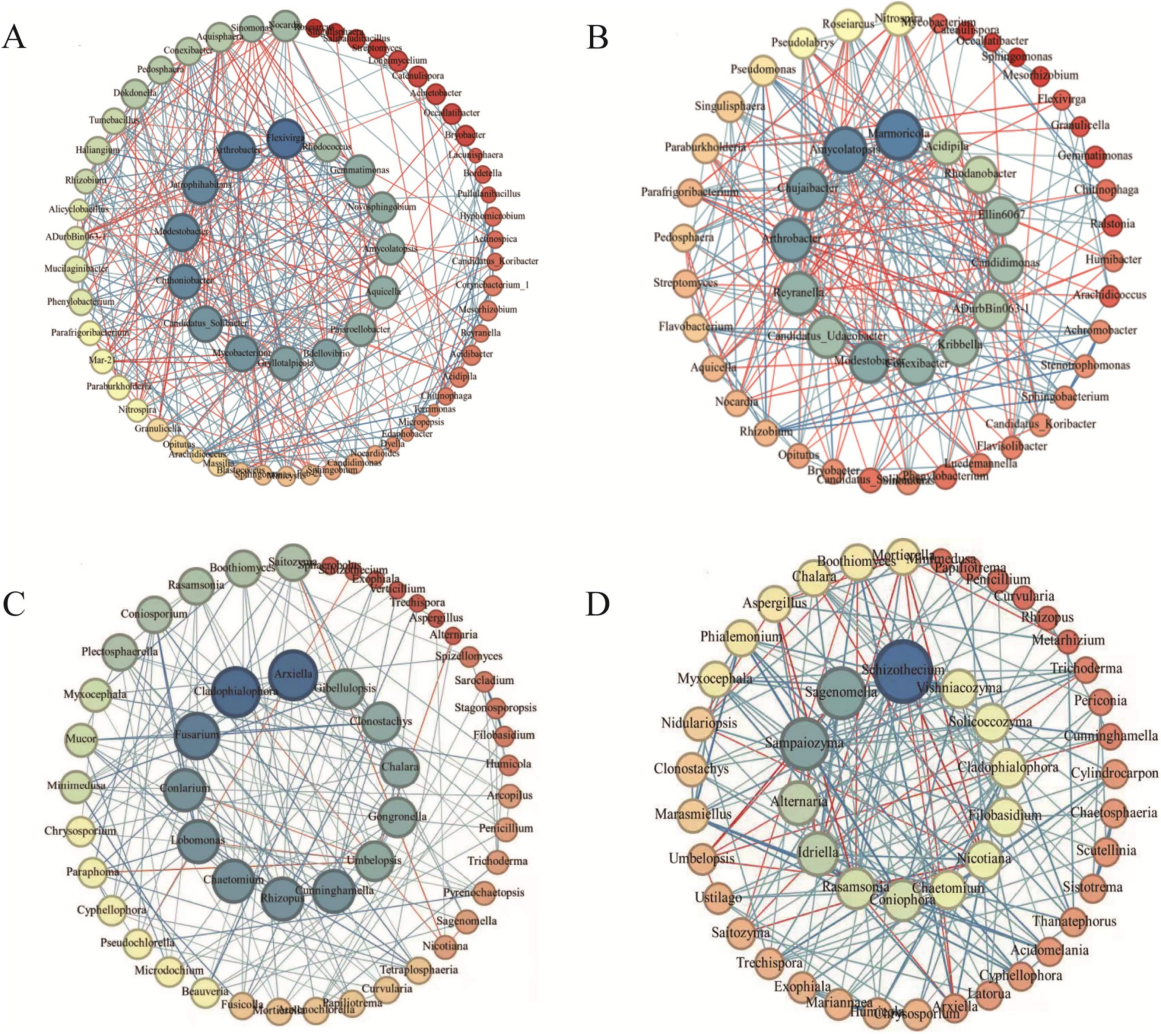
Soil microbial co-occurrence network analysis. The top 100 genera in relative abundance were selected for both bacteria and fungi. Pearson correlation analysis (*r* > 0.6, *p* < 0.05) was used to construct ecological network topologies. Red lines indicate positive correlations, while blue lines indicate negative correlations. Panels **(A, B)** represent bacterial networks in non-diseased and diseased soils, respectively, whereas panels **(C, D)** correspond to fungal networks in non-diseased and diseased soils, respectively. Node size is proportional to the relative abundance of each genus.

Comparison of bacterial keystone taxa revealed distinct differences between non-diseased and diseased soils. In non-diseased soils, the dominant bacterial genera included *Modestobacter, Jatrophihabitans, Flexivirga, Arthrobacter, Candidatus_Solibacter, Mycobacterium, Chthoniobacter,* and *Gemmatimonas* (Figure 5A). In diseased soils, the keystone bacterial genera shifted to *Marmoricola, Amycolatopsis, Chujaibacter, Arthrobacter,* and *Modestobacter* (Figure 5B), suggesting that infection by *Ralstonia solanacearum* reshaped the core bacterial network.

Similarly, fungal keystone taxa showed substantial shifts. In non-diseased soils, core fungal genera primarily included *Cladophialophora, Arxiella, Conlarium, Lobomonas, Chaetomium,* and *Fusarium* (Figure 5C). In diseased soils, the dominant fungal genera shifted to *Schizothecium, Sagenomella, Sampaiozyma, Rasamsonia, Chaetomium,* and *Cladophialophora* (Figure 5D). These findings indicate that infection by *R. solanacearum* altered most of the keystone fungal taxa in the rhizosphere microbial network (Table 2).

**Table 2:** Overview of the topological properties of soil microbial networks.

Network topological properties	Bacteria		Fungi	
	Non-disease	Disease	Non-disease	Disease
Nodes	69	79	51	48
Edges	346	340	189	249
Average degree	10.176	10.92	7.149	8.167
Average weighted degree	7.333	7.894	5.286	5.945
Network diameter	5	5	6	5
Graph density	0.152	0.223	0.155	0.174
Modularity	0.446	0.35	0.583	0.522
Average clustering coefficient	0.516	0.603	0.666	0.652
Average path length	2.446	2.149	2.583	2.414

### 3.7 Correlation analysis between core microbial genera and *Ralstonia solanacearum*

Core bacterial genera in both diseased and non-diseased soils were analyzed using a species abundance-based network approach. Pearson correlation coefficients were calculated to identify bacterial taxa associated with *Ralstonia*, the causal agent of tobacco bacterial wilt. In non-diseased soils, six bacterial genera were positively correlated with *Ralstonia*, while nine genera were negatively correlated. Among these, *Jatrophihabitans* exhibited the highest relative abundance (0.75%) and showed a significant positive correlation with *R. solanacearum* (Table 3). In diseased soils, five bacterial genera were positively correlated and nine negatively correlated with *Ralstonia*, with *Chujaibacter* displaying the highest relative abundance (2.72%) and a significant negative correlation with *R. solanacearum* (Table 4). Intersection analysis of significantly correlated genera in both diseased and non-diseased soils revealed that *Amycolatopsis* and *Arthrobacter* were consistently negatively correlated with *R. solanacearum*. For fungal communities, eleven genera in non-diseased soils were positively correlated with *Ralstonia*, and two were negatively correlated. Among these, *Umbelopsis* had the highest relative abundance (19.38%) and showed a positive correlation with *R. solanacearum* (Table 5). In diseased soils, three fungal genera were positively correlated and ten were negatively correlated with *Ralstonia*, with *Nicotiana* exhibiting the highest relative abundance (4.84%) and a negative correlation with *R. solanacearum* (Table 6). Intersection analysis of fungal genera indicated that *Chaetomium* and *Cladophialophora* were positively correlated with *Ralstonia* in non-diseased soils but negatively correlated in diseased soils, although these correlations were not statistically significant.

**Table 3:** Correlation between bacterial genera and *Ralstonia* in healthy (non-diseased) soil.

Pathogenic bacteria	Core bacterial genus	Correlation coefficient	Phylum	Relative mean abundance	*p*-value
*Ralstonia solanacearum*	*Modestobacter*	−0.669	*Actinobacteria*	0.260	0.057
	*Jatrophihabitans*	0.881	*Actinobacteria*	0.750	0.000
	*Flexivirga*	0.730	*Actinobacteria*	0.320	0.007
	*Arthrobacter*	−0.822	*Actinobacteria*	0.480	0.001
	*Candidatus_Solibacter*	−0.581	*Acidobacteria*	0.520	0.047
	*Mycobacterium*	0.641	*Actinobacteria*	0.610	0.025
	*Chthoniobacter*	−0.484	*Verrucomicrobia*	0.120	0.111
	*Gemmatimonas*	0.583	*Gemmatimonadetes*	0.360	0.047
	*Gryllotalpicola*	0.810	*Actinobacteria*	0.040	0.001
	*Pajaroellobacter*	−0.479	*Proteobacteria*	0.240	0.115
	*Amycolatopsis*	−0.307	*Actinobacteria*	0.070	0.033
	*Bdellovibrio*	−0.117	*Proteobacteria*	0.110	0.717
	*Novosphingobium*	−0.202	*Proteobacteria*	0.120	0.529
	*Nocardia*	0.294	*Actinobacteria*	0.090	0.354
	*Aquicella*	−0.589	*Proteobacteria*	0.210	0.044

**Table 4:** Correlation between bacterial genera and *Ralstonia* in diseased soil.

Pathogenic bacteria	Core bacterial genus	Correlation coefficient	Phylum	Relative mean abundance	*P*-value
*Ralstonia solanacearum*	*Marmoricola*	−0.377	*Actinobacteria*	0.690	0.228
	*Amycolatopsis*	−0.625	*Actinobacteria*	0.160	0.030
	*Chujaibacter*	−0.678	*Proteobacteria*	2.720	0.015
	*Arthrobacter*	−0.858	*Actinobacteria*	0.360	0.001
	*Modestobacter*	−0.453	*Actinobacteria*	0.100	0.139
	*Conexibacter*	−0.404	*Actinobacteria*	0.320	0.193
	*Reyranella*	0.411	*Proteobacteria*	0.270	0.185
	*Candidatus_Udaeobacter*	0.214	*Verrucomicrobia*	0.310	0.503
	*ADurbBin063-1*	0.920	*Verrucomicrobia*	0.250	0.001
	*Candidimonas*	−0.770	*Proteobacteria*	0.090	0.003
	*Ellin6067*	0.797	*Proteobacteria*	0.070	0.002
	*Kribbella*	0.812	*Actinobacteria*	0.060	0.001
	*Rhodanobacter*	−0.864	*Proteobacteria*	1.250	0.001
	*Acidipila*	−0.540	*Acidobacteria*	0.310	0.070

**Table 5:** Correlation between fungal genera and *Ralstonia* in healthy (non-diseased) soil.

Pathogenic bacteria	Core bacterial genus	Correlation coefficient	Phylum	Relative mean abundance	*P*-value
*Ralstonia solanacearum*	*Cladophialophora*	0.780	*Ascomycota*	0.230	0.063
	*Arxiella*	0.772	*Ascomycota*	0.060	0.003
	*Conlarium*	−0.198	*Ascomycota*	1.890	0.537
	*Lobomonas*	−0.146	*Chlorophyta*	0.010	0.650
	*Chaetomium*	0.701	*Ascomycota*	0.060	0.051
	*Fusarium*	0.911	*Ascomycota*	0.220	0.000
	*Rhizopus*	0.833	*Mucoromycota*	0.090	0.001
	*Gongronella*	0.702	*Mucoromycota*	2.720	0.011
	*Chalara*	0.623	*Ascomycota*	0.220	0.031
	*Clonostachys*	0.926	*Ascomycota*	0.090	0.000
	*Cunninghamella*	0.876	*Mucoromycota*	0.070	0.000
	*Gibellulopsis*	0.623	*Ascomycota*	0.140	0.030
	*Umbelopsis*	0.344	*Mucoromycota*	19.380	0.273

**Table 6:** Correlation between fungal genera and *Ralstonia* in diseased soil.

Pathogenic bacteria	Core bacterial genus	Correlation coefficient	Phylum	Relative mean abundance	*P*-value
*Ralstonia solanacearum*	*Schizothecium*	0.159	*Ascomycota*	0.520	0.621
	*Sagenomella*	−0.371	*Ascomycota*	0.160	0.235
	*Sampaiozyma*	−0.198	*Basidiomycota*	0.050	0.538
	*Alternaria*	−0.198	*Ascomycota*	0.600	0.536
	*Idriella*	−0.122	*Ascomycota*	0.030	0.706
	*Rasamsonia*	−0.157	*Ascomycota*	0.110	0.627
	*Coniophora*	−0.067	*Basidiomycota*	0.030	0.837
	*Chaetomium*	−0.343	*Ascomycota*	0.100	0.274
	*Nicotiana*	−0.228	*Anthophyta*	4.840	0.475
	*Filobasidium*	−0.379	*Basidiomycota*	0.070	0.224
	*Cladophialophora*	−0.047	*Ascomycota*	0.190	0.884
	*Solicoccozyma*	0.193	*Basidiomycota*	0.050	0.548
	*Vishniacozyma*	0.106	*Basidiomycota*	0.040	0.743

### 3.8 Environmental factors and key microbial taxa

**Figure 6:**
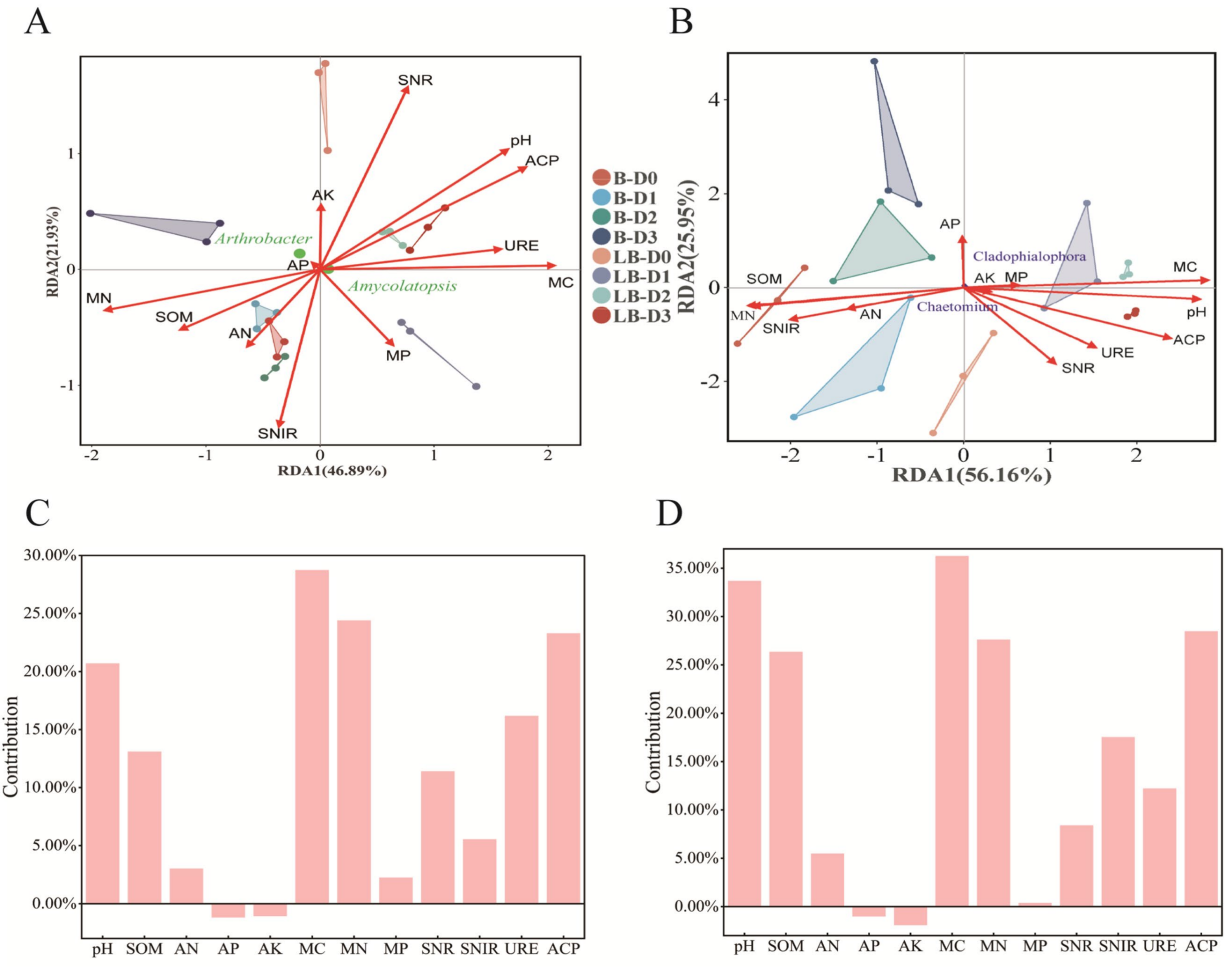
Analysis of environmental factors and key microbial genera. Key microbial genera in bacterial and fungal communities were combined with OTU data matrices for redundancy analysis (RDA) and variance partitioning analysis (VPA). In the RDA plots, arrows represent environmental factors, with longer arrows indicating a stronger influence on microbial community distribution. In the VPA plots, larger values indicate a greater effect of the environmental factor, while negative values indicate no significant effect. Panels **(A, C)** correspond to bacterial RDA and VPA, respectively, whereas panels **(B, D)** correspond to fungal RDA and VPA, respectively.

Environmental factors acted as major drivers of soil microbial community composition, with distinct key taxa showing specific responses to different factors. In the bacterial community, vectors representing nitrate reductase activity (SNR), pH, acid phosphatase activity (ACP), microbial biomass carbon (MC), microbial biomass nitrogen (MN), and nitrite reductase activity (SNIR) were relatively long (Figure 6A), indicating strong associations. Specifically, pH, ACP, and MC promoted the enrichment of *Amycolatopsis*, while MN and soil organic matter (SOM) favored *Arthrobacter*. Variance partitioning analysis revealed that MC (28.74%), MN (24.40%), ACP (23.29%), and pH (20.70%) contributed substantially to bacterial community variation (Figure 6C).

In the fungal community, vectors for SOM, MN, MC, pH, and ACP were prominent (Figure 6B), promoting the enrichment of *Chaetomium* and *Cladophialophora*. Variance partitioning further indicated that pH (33.70%), MC (36.28%), ACP (28.49%), MN (27.63%), and SOM (26.36%) were major contributors to fungal community variation (Figure 6D).

## 4 Discussion

Nitrogen is an essential nutrient for plant growth, influencing not only plant development but also the occurrence of plant diseases (Ding et al., 2021). Previous studies have indicated that low nitrogen levels can reduce the incidence of tobacco bacterial wilt, caused by *Ralstonia* spp. (Wang et al., 2022b; Shen et al., 2022), which aligns with the present study, where decreasing nitrogen levels corresponded with reductions in both disease incidence and severity. Our results also showed that diseased soils harbored significantly higher *Ralstonia* populations compared to non-diseased soils, consistent with previous findings. The presence of low levels of *Ralstonia* in the rhizosphere of healthy plants suggests that pathogen presence alone does not determine disease occurrence; rather, disease development is likely influenced by a combination of rhizosphere factors (Wei et al., 2018). Furthermore, reductions in nitrogen application significantly decreased *Ralstonia* copy numbers, indicating that nitrogen fertilization can affect pathogen abundance, possibly via alterations in soil microbial community structure and diversity, which in turn influence pathogen dynamics (Maaroufi et al., 2015). Notably, some studies have reported that low nitrogen can exacerbate plant disease, highlighting the complex relationship between nitrogen utilization and disease progression (Fagard et al., 2014). In addition to nitrogen levels, nitrogen forms also play critical roles in disease modulation. Nitrate tends to promote *R. solanacearum* colonization and stem infection, whereas ammonium fertilizers often suppress pathogen activity by increasing soil pH. This highlights the need to consider both nitrogen rates and nitrogen forms in future studies (Dalsing and Allen, 2014; RuiFeng et al., 2024).

Soil nutrients are closely linked to plant health and play critical roles in soil-borne disease development (Mahmood, 2013). The incidence of tobacco bacterial wilt is influenced by multiple environmental factors, including temperature and pH, as well as soil compaction and physicochemical degradation, which can exacerbate disease severity (Bittner et al., 2016; Zhang et al., 2020). Previous research has shown that rhizosphere soils of diseased plants often have lower available phosphorus but higher pH, organic matter, alkaline nitrogen, and available potassium (Zhang et al., 2020). In contrast, our study found that diseased soils had lower pH, organic matter, and hydrolyzable nitrogen but higher available phosphorus and potassium than non-diseased soils. Elevated available nutrients in diseased soils may be due to site-specific conditions or nutrient accumulation following plant infection, resulting in weakened plants more susceptible to pathogen invasion (Li P. et al., 2023; Zhang Y. et al., 2022).

Microbial biomass carbon (MC) and nitrogen (MN) are key indicators of soil quality and fertility (Ashraf et al., 2020; Bruni et al., 2025), reflecting microbial activity and involvement in nearly all soil biochemical processes (Moore et al., 2000; Rice et al., 1997; Nyamwange et al., 2021). Microbial biomass phosphorus (MP) plays a crucial role in the bioavailability of phosphorus for plants (Chen et al., 2019; Peng C. et al., 2021). Soil microbial biomass N closely correlates with soil nitrogen availability, and moderate nitrogen application can enhance microbial biomass C and N (He et al., 2024; Huang et al., 2019; Li W. et al., 2023). Consistently, our study observed an initial increase followed by a decrease in microbial biomass C with reduced nitrogen, while diseased soils exhibited higher MC but lower MN than non-diseased soils, suggesting reduced nitrogen utilization following infection (Qi et al., 2020; Li S. et al., 2021).

Nitrogen application also influences soil pH and chemical properties. Previous studies indicate that increasing nitrogen lowers soil pH and increases nitrate and ammonium concentrations while decreasing available phosphorus and potassium (He et al., 2021; Zhao et al., 2022; Zeng et al., 2025). In our study, decreasing nitrogen in non-diseased soils led to a slight increase in pH and reductions in organic matter, hydrolyzable nitrogen, available phosphorus, and potassium. Correlation analysis revealed a significant negative relationship between pH and *Ralstonia* abundance, suggesting that higher soil pH under lower nitrogen may be critical in preventing disease (Liu et al., 2020; Liu X. et al., 2024). In diseased soils, decreasing nitrogen reduced organic matter but increased available phosphorus and potassium, likely due to rhizosphere alterations after infection (Peng Y. et al., 2021; Cao et al., 2024).

Soil enzyme activities play pivotal roles in nutrient transformation and availability, reflecting plant nutrient uptake and utilization (Neemisha and Sharma, 2022; Piotrowska-Długosz, 2019). In this study, urease, acid phosphatase, and nitrate reductase activities were higher in diseased rhizosphere soils than in non-diseased soils, potentially reflecting microbial responses to pathogen stress to aid plant resistance (Hou et al., 2021; Chi et al., 2025). Nitrogen levels can modulate enzyme activities, where moderate N enhances activity but excessive N may suppress it (Liu et al., 2020). In non-diseased soils, urease and acid phosphatase activities peaked under 20% nitrogen reduction, suggesting that appropriate nitrogen levels optimize enzyme-mediated nutrient cycling. Activities of nitrate reductase increased and nitrite reductase decreased with declining nitrogen levels, consistent with previous observations (Barneix et al., 1984; Wan et al., 2025).

Rhizosphere microbial communities are crucial for plant health and disease suppression (Ma et al., 2025; Shi et al., 2021). Bacterial and fungal diversity patterns in diseased soils differed from non-diseased soils: Shannon and Simpson indices for bacteria were higher in diseased soils under similar nitrogen conditions, and fungal richness and diversity were markedly higher in diseased soils. This may result from plant metabolic disturbances post-infection, leading to altered root exudates or endophyte recruitment, thereby increasing microbial diversity (Ahmed et al., 2022; Fracchia et al., 2024). Reduced nitrogen led to a decrease-then-increase trend in bacterial diversity in non-diseased soils, whereas fungal richness increased under 30% nitrogen reduction, indicating nitrogen impacts microbial community structure and diversity. PCoA analyses showed gradual separation of bacterial and fungal communities with decreasing nitrogen, particularly among treatments in non-diseased soils, confirming that nitrogen level and disease status shape microbial communities.

At the phylum level, dominant bacterial taxa included Actinobacteria, Proteobacteria, and Acidobacteria, while dominant fungal phyla were Ascomycota, Mucoromycota, and Basidiomycota, consistent with previous studies (Liu et al., 2025; Gao et al., 2017). At the genus level, *Chujaibacter*, *Sphingomonas*, and *Bryobacter* were prevalent among bacteria, while *Mortierella*, *Saitozyma*, and *Trechispora* dominated fungi. Notably, *Sphingomonas* and *Bryobacter*, considered beneficial taxa, showed increased abundance in diseased soils, potentially improving rhizosphere nutrient availability and plant resistance (Wang et al., 2024; ZHANG et al., 2023). Fungal genera such as *Mortierella* and *Saitozyma* also increased under reduced nitrogen, enhancing nutrient transformation and stress resistance (Liu et al., 2025; Ozimek and Hanaka, 2020). The PCoA results showed no clear separation between the conventional N treatment and the 10% N reduction in disease-free soils, suggesting that microbial communities may exhibit a threshold or non-linear response to N reduction. Small decreases in N input may not exceed the adaptive capacity of the microbial community, resulting in minimal structural changes. Significant shifts in community composition are more likely to occur only when N reduction surpasses a certain threshold, consistent with previous studies reporting non-linear and threshold effects of N availability on soil microbial communities (Chen et al., 2024).

Ecological network analysis revealed more complex but less stable microbial interactions in diseased soils, with reduced network diameter, density, and path length, corroborating previous findings that disease alters core microbial taxa and community stability (Wen et al., 2020; Wang H. et al., 2022). Correlation analyses identified *Arthrobacter* and *Amycolatopsis* as key bacterial genera negatively associated with *Ralstonia*, both known for biocontrol potential and plant growth promotion (Ramlawi et al., 2021; Kim et al., 2023; Sriwati et al., 2023). Reduced nitrogen increased their abundance, indicating that nitrogen management can modulate rhizosphere beneficial microbes and disease incidence. However, the specific suppression mechanisms—such as antibiotic secretion, nutrient competition, or induced systemic resistance—were not experimentally validated in this study. Future research incorporating *in vitro* confrontation assays, metabolite profiling, or expression analysis of functional genes (e.g., PKS/NRPS pathways) will be valuable for mechanistic elucidation.

Under reduced nitrogen conditions, the decline in *R. solanacearum* abundance likely results from a combination of nutrient limitation, mitigation of soil acidification, and increased competition from enriched beneficial microbes, collectively fostering a disease-suppressive soil environment. RDA and variance partitioning analyses highlighted that soil properties (pH, MC, MN, SOM) and enzyme activities (ACP) significantly influence bacterial and fungal community shifts, correlating with core genera (*Arthrobacter* and *Amycolatopsis*), consistent with prior studies (Deng et al., 2019; Yang et al., 2021). In particular, soil pH and microbial biomass carbon showed strong regulatory effects on microbial niche differentiation. Higher pH levels promoted beneficial Actinobacteria, while elevated microbial biomass carbon enhanced microbial competition intensity, limiting pathogen ecological niches. These synergistic interactions demonstrate how soil physicochemical properties regulate microbial assembly processes and ultimately influence disease outcomes (Barka et al., 2016; Schlatter et al., 2017). These findings suggest that lower nitrogen levels reshape rhizosphere nutrient and microbial environments, thereby impacting tobacco bacterial wilt development. From an ecological perspective, reduced nitrogen inputs may shift rhizosphere interactions toward a disease-suppressive state via several linked mechanisms. Lowering N inputs can alleviate soil acidification and thus create pH conditions less favorable for *R. solanacearum* growth and colonization. Indeed, acidic soils tend to favor Ralstonia, while higher pH suppresses the pathogen and enriches antagonistic taxa (Li et al., 2017). In addition, N limitation intensifies competition for nutrients and niches among soil microbes, and promotes the relative dominance of Actinobacteria and other antagonists that produce antimicrobial secondary metabolites (Boubekri et al., 2022). Together, these pH-mediated effects, resource competition and antagonist enrichment can synergize to form a disease-suppressive microbiome that limits *R. solanacearum* success (Jayaraman et al., 2021).

In summary, this study systematically explored the effects of nitrogen levels on rhizosphere soil physicochemical properties, enzyme activities, microbial community structure, and their relationships with tobacco bacterial wilt. However, limitations remain: the effects of nitrogen management may vary across ecological zones and cropping systems; the mechanisms linking nitrogen cycling to disease suppression require further clarification; and the differential impacts of nitrogen forms on microbial communities and disease were not examined. Future studies should employ long-term, multi-site trials across soil types and climates to elucidate nitrogen-driven ecological mechanisms influencing bacterial wilt, thereby informing precision nitrogen management and sustainable disease control strategies. As the present study was conducted in a single yellow-soil site, future multi-site field experiments across diverse soil types and climatic regions are needed to validate the general applicability of nitrogen-reduction strategies for bacterial wilt management. In addition, the initial soil microbial community prior to treatment application was not characterized in the present study. Although all plots were located within the same field, managed under identical agronomic practices, and randomly assigned to treatments to minimize baseline heterogeneity, we cannot completely exclude the possibility that pre-existing spatial variation contributed to part of the observed differences among treatments. Future studies incorporating baseline microbial sampling before fertilization would help to better disentangle treatment effects from initial community structure. Because the experiment was conducted over a single growing season, interannual variability in disease incidence and microbial responses could not be assessed. Long-term or multi-season field trials will be necessary to verify the stability of the observed nitrogen-reduction effects on bacterial wilt suppression.

## 5 Conclusion

This study systematically elucidated the regulatory mechanisms of basal nitrogen reduction on the occurrence of tobacco bacterial wilt and its rhizosphere microecology. The results demonstrated that under reduced nitrogen conditions, soil pH, organic matter, microbial biomass carbon, and acid phosphatase activity acted as key drivers shaping the rhizosphere microbial community. Nitrogen reduction modulated microbial community structure, enhanced *α*-diversity and network stability, and promoted the enrichment of beneficial bacterial genera such as *Arthrobacter* and *Amycolatopsis*, thereby significantly suppressing *Ralstonia* abundance and reducing the incidence of tobacco bacterial wilt. In summary, moderate nitrogen reduction effectively improved soil physicochemical properties and microbial community composition, enhanced tobacco resistance to disease, and inhibited bacterial wilt occurrence. These findings provide a scientific basis for optimizing nitrogen fertilizer management, enabling sustainable disease control and supporting the sustainable production of tobacco through nutrient-driven strategies for bacterial wilt management. Nevertheless, as the current study was limited to a single yellow-soil site, further experiments across multiple soil types and climatic environments are essential to determine the wider applicability of nitrogen-reduction approaches for bacterial wilt control.

## Data Availability

The datasets presented in this study can be found in online repositories. The names of the repository/repositories and accession number(s) can be found in the article/Supplementary material.
