# Supplementary material for: Nitrogen reduction in basal fertilization enhances soil physicochemical properties and reshapes microbial community structure to alleviate tobacco bacterial wilt
Source: Front Microbiol. 2026 Jan 16;16:1704525. doi: 10.3389/fmicb.2025.1704525 (PMC12858187; doi:10.3389/fmicb.2025.1704525)
Supplement: Supplementary file 1 [file Table_1.DOC]

Supplementary Material

Figure Captions

**Figure S1. Standard curve of *Ralstonia solanacearum*.**
The standard curve was generated to quantify *Ralstonia solanacearum* in soil samples, based on qPCR using known concentrations of bacterial DNA.


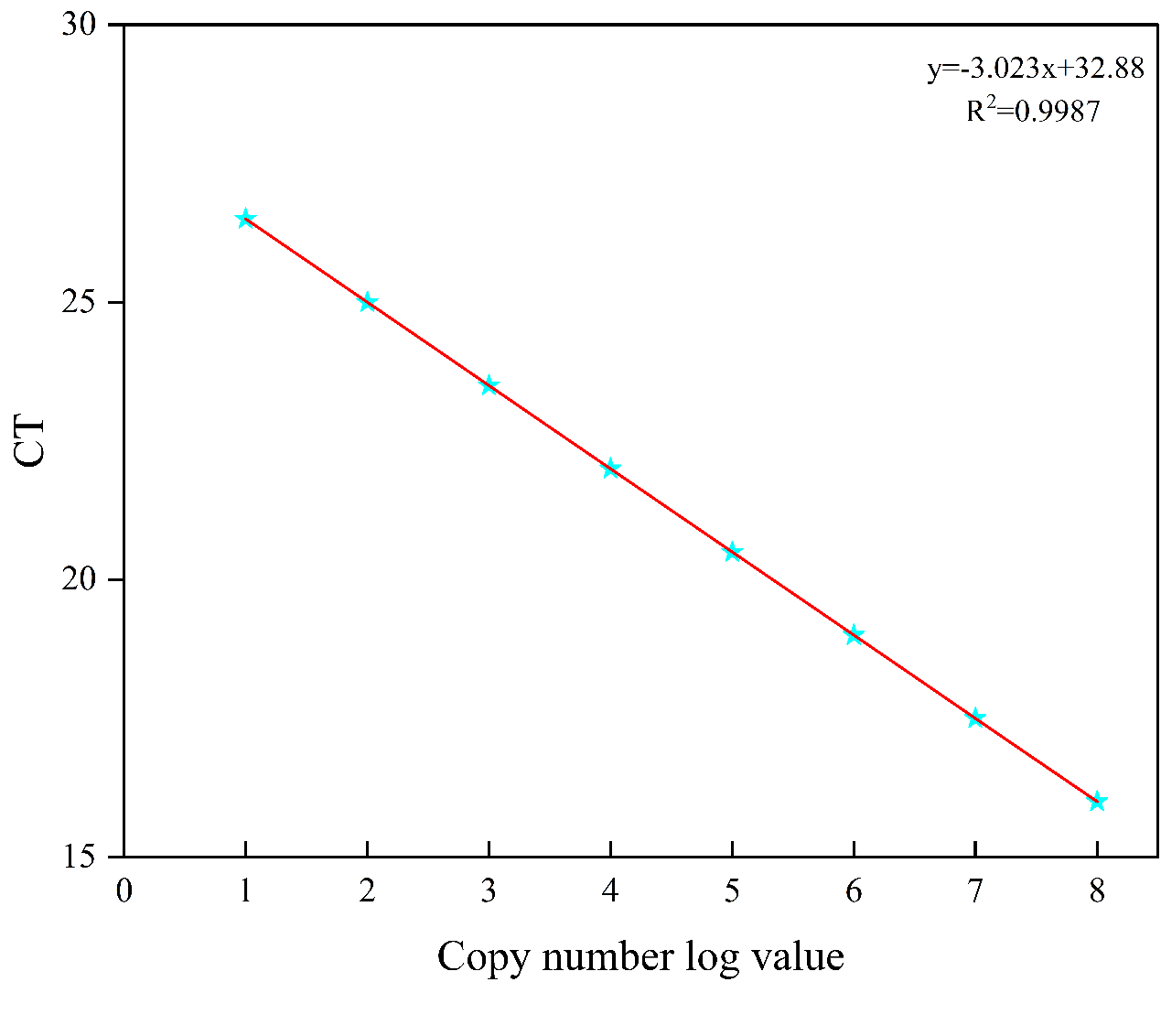


Fig.S1

**Table Caption**

**Table S1. Disease incidence and severity index under different basal nitrogen reduction treatments.**
Disease incidence (%) and disease severity index of tobacco plants were measured under varying levels of basal nitrogen reduction. Values are presented as mean ± standard deviation. Different letters indicate statistically significant differences among treatments (p < 0.05). Treatments included two soil types, non-diseased soil (B) and diseased soil (LB), combined with four basal nitrogen (N) application levels. For non-diseased soil, treatments were designated as B-D0 (conventional fertilization, CK, 75 kg N·hm⁻²), B-D1 (10% basal N reduction, 67.5 kg N·hm⁻²), B-D2 (20% basal N reduction, 60 kg N·hm⁻²), and B-D3 (30% basal N reduction, 52.5 kg N·hm⁻²). For diseased soil, corresponding treatments were LB-D0 (CK, 75 kg N·hm⁻²), LB-D1 (10% reduction, 67.5 kg N·hm⁻²), LB-D2 (20% reduction, 60 kg N·hm⁻²), and LB-D3 (30% reduction, 52.5 kg N·hm⁻²).

**Table S2. Copy number of *Ralstonia solanacearum* in healthy and diseased soils.**
The abundance of *Ralstonia solanacearum* was quantified in soil samples from healthy and diseased plots. Values are presented as mean ± standard deviation. Different letters indicate statistically significant differences between treatments (p < 0.05).

**Table S1**

| Treatment | Disease Incidence (%) | Disease Severity Index |
| --- | --- | --- |
| LB-D0 | 11.93 a | 5.72 a |
| LB-D1 | 9.86 b | 4.80 ab |
| LB-D2 | 7.05 c | 3.35 c |
| LB-D3 | 7.84 c | 3.20 c |

**Table S2**

|  | Treatment | *R. solanacearum* Copy Number (copies/g) |
| --- | --- | --- |
| Non-disease | B-D0 | 5.37±0.06d |
|  | B-D1 | 2.07±0.95d |
|  | B-D2 | 0.31±0.05d |
|  | B-D3 | 0.54±0.07d |
| Disease | LB-D0 | 3631.29±730.96a |
|  | LB-D1 | 1143.41±269.51b |
|  | LB-D2 | 679.99±6.63bc |
|  | LB-D3 | 600.96±26.72c |
